# Advancing Cancer Workforce Capacity for American Indians and Alaska Natives: The Development of a Validated System to Optimize Trainee Participation and Outcome Tracking

**DOI:** 10.3390/ijerph21060752

**Published:** 2024-06-08

**Authors:** Kelly A. Laurila, Laurie D. Rogers, Celina I. Valencia, Naomi Lee, Hendrik de Heer, Jennifer W. Bea, Jani C. Ingram, Francine C. Gachupin

**Affiliations:** 1 Center for Health Equity Research, Northern Arizona University, Flagstaff, AZ 86011, USA; 2 Office of the Vice President for Research, Northern Arizona University, Flagstaff, AZ 86011, USA; laurie.rogers@nau.edu; 3 Department of Family and Community Medicine, University of Arizona, Tucson, AZ 85716, USA; celina@arizona.edu (C.I.V.); fcgachupin@arizona.edu (F.C.G.); 4 Department of Chemistry and Biochemistry, Northern Arizona University, Flagstaff, AZ 86011, USA; naomi.lee@nau.edu (N.L.); jani.ingram@nau.edu (J.C.I.); 5 Department of Health Sciences, Northern Arizona University, Flagstaff, AZ 86011, USA; dirk.deheer@nau.edu; 6 Department of Health Promotion Sciences, University of Arizona, Tucson, AZ 85724, USA; jbea@uacc.arizona.edu

**Keywords:** trainee tracking, cancer workforce capacity, American Indian and Alaska Native, National Student Clearinghouse, cancer health disparities, social determinants of health

## Abstract

Although American Indian and Alaska Native (AIAN) students are the most underrepresented group in the U.S. in biomedical and health sciences relative to population size, little is known about long-term research education programs and outcome tracking. For over 20 years, the Partnership for Native American Cancer Prevention (NACP) has been supported under the National Cancer Institute’s (NCI)-funded Comprehensive Partnerships to Advance Cancer Health Equity (CPACHE) program. Programming included hands-on mentored research and an array of development opportunities. A validated tracking system combining participation records, institutional records, and enrollment/degree attainment from the National Student Clearinghouse documents outcomes. Collectively (2002–2022) NACP engaged 367 AIAN trainees, of whom 237 individuals earned 220 bachelors, 87 masters, and 34 doctoral/professional degrees. Approximately 45% of AIAN doctoral recipients are currently engaged in academic or clinical work, and 10% in industry or tribal leadership. A total of 238 AIAN students participated in mentored research, with 85% demonstrating strong outcomes; 51% attained a degree, and 34% are currently enrolled. Implementation of a robust tracking system documented acceleration in degree attainment over time. Next steps will evaluate the most impactful training activities on student outcomes.

## 1. Introduction

American Indian and Alaska Native (AIAN) students are the single most underrepresented racial group in the biomedical and health sciences relative to population size [1]. Disparities in educational attainment are more pronounced at each successive level, with 0.4% AIAN enrollment in graduate STEM programs in 2021 [2]. The most recent (2021) NSF National Center for Science and Engineering Statistics Survey of Earned Doctorates (NCSES) showed that across the United States (U.S.), in a single year, only 64 AIANs earned a doctoral degree in science or engineering [3]. Native American applicant rates are so low they are excluded from National Institutes of Health (NIH) Research Project Grant data reporting. In fact, the NIH’s March 2023 *Analyses of Demographic-Specific Funding Rates for Type 1 Research Project Grants and R01-Equivalent Applications* did not include Native Americans, noting the sample size was too small, citing concerns about identifying applicants (<12) [4].

Term use explanation: According to the Office of Management and Budget (OMB), American Indian/Alaska Native refers to a person having origins in any of the original peoples of North and South America, including Central America, and who maintains tribal affiliation or community attachment. Native American is a social term that is more inclusive and includes urban Natives. Often American Indian and Native American are used interchangeably. The Native American populations across the United States are recognized collectively as Indian Country. First Peoples from around the world are known as Indigenous.

To address the significant disparities among individuals from AIAN, African American, and Latinx populations, henceforth referred to as underrepresented (UR) communities, the NIH and other federal funding agencies such as the National Science Foundation have strategically invested in training programs to expand workforce capacity and diversity [5,6,7]. At the NIH level, the Chief Officer for Scientific Workforce Diversity (COSWD) was appointed by the NIH Director in 2014 to serve as an advisor to catalyze concerted efforts across the 27 NIH Institutes and Centers and NIH-supported institutions for diversity, equity, inclusion, and accessibility [8,9]. The COSWD is tasked with leading NIH efforts to diversify the nation’s scientific workforce, which includes expanding recruitment and retention and reporting directly to the NIH Director [10]. In 2015, the NIH founded the Tribal Health Research Office (THRO). The THRO 2019–2023 Strategic Plan focuses on four goals, with goal 2 aimed at building research capacity for AIAN communities. Embedded in the goal is increasing the number of AIAN students who pursue health and biomedical research careers [11]. At the National Cancer Institute (NCI), the Center to Reduce Cancer Health Disparities (CRCHD), established in 2001, works on the three programmatic areas of cancer health disparities research, diversity training, and outreach [5]. The programmatic focus of CRCHD reflects the notion that the successful reduction of cancer health disparities is inextricably linked to the achievement of a diverse and inclusive biomedical research workforce [5]. Within the CRCHD diversity training portfolio is the Continuing Umbrella of Research Experiences (CURE), established in 1999 to strengthen the STEM pipeline for UR populations.

The portfolio also includes the Comprehensive Partnership to Advance Cancer Health Equity (CPACHE) training program, which engages diverse scientists tailored for the local community [5]. Collectively, CPACHE targets cancer research, cancer education and cancer outreach to advance cancer health equity. Each CPACHE program is a partnership between institutions serving underserved health disparity populations and underrepresented students (ISUPS) and NCI-designated cancer centers. The CPACHE primary goals centered around workforce development are to *increase the cancer research and cancer research education capacity of the ISUPSs; increase the number of students and investigators from underrepresented populations engaged in cancer research; and increase the number of investigators and students conducting cancer health disparities research* [12]. Within the context of a CPACHE-funded program, the Partnership for Native American Cancer Prevention (NACP), this paper outlines key considerations and strategies for the development and implementation of a trainee tracking system to support federally funded workforce diversity programs with limited resources to document, track, and disseminate program outcomes for UR trainees. In addition, we provide data on degree completion and professional positions of AIAN trainees over a 20-year period, contributing to the minimal literature of career outcomes among AIAN trainees in STEM fields.

*The Partnership for Native American Cancer Prevention*: NACP is a funded CPACHE grant between the University of Arizona’s NCI-designated comprehensive Cancer Center (UACC) and Northern Arizona University (NAU) as the partner institution serving underserved health disparity populations and underrepresented students (ISUPS). NACP was established in 2002 with the overarching goals to *reduce the cancer burden within the Native American population through research and community engagement; expand the number of Native American investigators working in cancer research; and increase the total number of investigators focused on cancer health disparities within the Native American communities of Arizona*. Through the longstanding CPACHE partnership, historic engagement, and health and cancer-focused career successes, we reached trainees representing 45 tribal nations, including 12 of the 22 federally recognized tribes in Arizona and at least 33 other tribal nations beyond Arizona.

In its nascent years, identifying Native American students to participate was a challenge, as typical strategies for recruiting students to participate in research activities such as websites, listservs, and announcements in classrooms were found to be less successful for Native American students compared to other student groups. A more effective approach was personal invitations for Native American students through NACP’s expanding networks, institutional databases, and referrals from instructors and already engaged students. Further, a breadth of activities, including Indigenous scientist role model speaker series, local conferences, events, and engagement with Native American serving student groups, further allowed the partnership to increase its reach.

*A brief history of CPACHE student engagement strategies*: Given the CPACHE program’s 20+ year history, it is important to highlight significant changes in programming through time. Table 1 outlines key features for student engagement required by the funding agency based on each funding opportunity relevant to NACP funding and the specific focus of this paper. Two key items include the emphasis on sustainability (an expectation that CPACHE programming should result in competitive cancer training programs such as T32, R25, F31, and K12 mechanisms) and a shift from a training program to a distinct research education focus in 2018 (implying that partnerships should seek additional external funding sources to fund/sustain research training endeavors).

*Overview and classification of NACP student engagement:* Since 2002, funding agency shifts in the focus of CPACHE efforts (from research training to research education, Table 1) have allowed the NACP student engagement activities to evolve. Table 2 provides a collective overview of the types of engagement, research training, and education activities that the partnership has collectively engaged in over the past 20+ years and selects examples of programming. Given the array of partnership opportunities, the evaluation team categorized activities by two broad categories, i.e., mentored research and professional development opportunities.

*The importance of advisory boards to provide critical feedback:* CPACHE programs are required to include an Internal Advisory Committee (IAC) and a Program Steering Committee (PSC), a national group of experts that advises partnerships. The IAC and PSC’s role in partnership direction and evaluation is critical to program advancement and identification of areas of concern. A key example of the importance of the PSC critically evaluating NACP occurred in 2011, when the PSC recommended that NACP develop an outcome tracking system to evaluate student engagement to help determine programmatic success. With the addition of an internal evaluation team in 2009, serving the entire partnership, housed at NAU (the ISUPS), NACP started tracking basic participation and documenting which students were engaged in research projects and summer training opportunities. Given that many ISUPS institutions may have less infrastructure and funding, CPACHE resources tend to be centered on research; the development of evaluation solutions that do not require large amounts of funding to establish and manage are critical. It is imperative to note that training programs require long-term investment and tracking to document outcomes to see whether the program is reaching its training/education goals. While CPACHE programs span the continuum from undergraduate through early career faculty, the focus of this paper is tracking outcomes of students from undergraduate through doctoral programs.

*Existing literature for developing systems to implement trainee tracking and outcome systems*:

Student tracking (linked data such as enrollment and graduation data, as well as longitudinal outcome data) is an essential metric in educational settings that can have wide-ranging implications regarding student success and programmatic improvement [13]. Higher education institutions regularly utilize linked data such as the National Student Clearinghouse (NSC) and institutional records to document academic program success. However, strategies for developing a linked and validated student tracking approach, specifically concerning research training programs, are not well documented in the literature. There are a few examples of linked and validated student tracking approaches out of Australia, primarily using institutional data to inform enrollment and graduation of medical professionals to inform surveys to document career trajectory [14,15]. We find in the U.S. literature documenting trainee tracking that instead of relying on institutional data or a linked data source like NSC, surveys are often used to gather data about enrollment and graduation, relying on individual respondents to provide information about their current status [16,17,18]. The New Mexico State University/Fred Hutch Cancer Center CPACHE partnership relies on gathering student emails through personal communication and social media; they do not use institutional data or another linked data source such as NSC and have called their attempts at maintaining an updated list of student email addresses an “ongoing effort” [16]. Long-term student outcome tracking is difficult to do. Institutional data systems are varied (which system, if any, is supported by the institution), career outcome data are incomplete (many institutions rely on social media, e.g., LinkedIn, Facebook, etc., to update their records), and technical challenges and variability in systems add further challenges [19]. While longitudinal outcome tracking is difficult, we know that there is immense value in the resulting data. The New Mexico State University/Fred Hutch Cancer Center CPACHE partnership emphasized the importance of the data obtained from tracking long-term outcomes while highlighting their program’s underrepresented student (graduate and undergraduate) achievements [16].

CareerTrac was established by the NIH in 2006 to increase the NIH’s effectiveness in evaluating health research training programs, including R25, K, and F awards [20]. NIH only recently (2021) implemented CareerTrac as a requirement for the CPACHE program. CareerTrac focuses primarily on productivity-focused outcomes such as publications, grants, honors/awards, and education. CareerTrac reports can be exported and added to an annual NIH Research Performance Progress Report (RPPR). Given that many CPACHE partnerships are over 20 years old, the adoption of CareerTrac has limitations. The system is not designed for retrospective tracking. Thus, long-standing sites need to use other modalities for tracking long-term outcomes from the life of the partnership to support competitive renewals (evidence of success) and inform data-driven decision-making. The implementation of CareerTrac for CPACHE sites provided clear parameters for tracking; criteria are as follows: *Trainees must have a hands-on role in the partnership-sponsored research project or Core for the purpose of fostering competitiveness that will lead to research independence. In addition, trainees must fall into one of these two categories: (1) those who are financially supported on a partnership-sponsored research project or Core for the purpose of acquiring research-related knowledge and/or skills; OR (2) those who may not be named in the budget or financially supported but are part of a research team in a project funded by the grant in order to acquire research-related knowledge and/or skills* [21]. The NACP tracks postdocs and early-stage investigators through separate mechanisms not discussed in this paper, which centers on individuals in undergraduate through trainees in doctoral/professional programs.

## 2. Methods

*Utilization-focused evaluation framework:*

A key feature of the literature discussed above is evaluation data sources and the ability to produce tracking relevant outcomes that are utilization-focused. The NACP evaluation team applies a utilization-focused framework (U-FE), which centers on utility as a throughline connecting the design process to the use of the findings to support the program’s ability to align programmatic findings with the CPACHE program goals. Successful implementation of the U-FE acknowledges a “complex, dynamic, and iterative system of relationships with the various elements and steps interacting” [22]. The NACP evaluation system features a feedback loop that involves continuously working with the partnership to improve and enhance the use of the evaluation. Engaging leadership (the primary users) in the design of the outcome tracking system increases utilization by program leadership by engaging with the findings [22]. Engagement started in the design phase to cultivate a shared vision for the primary metrics in the database. Priority evaluation questions connect to the teams ability to speak to NACP’s progress toward CPACHE training and workforce goals. Designing the database output included an iterative process, where initial results were shared with leadership and the evaluation team received feedback about important elements and the ability to show change through time and ultimately speak to the NACP’s effectiveness of the program [22].

*Development of a retrospective process to implement a trainee tracking system:* Driven by the PSC recommendation, the evaluation team set out to develop a systematic approach to participation and outcome tracking. The team worked with partnership leadership to identify institutional sources, including a conversation with the NAU Institutional Research and Analysis Office and the Human Subjects review board. This resulted in the submission of a Human Subject Determination form to confirm that student tracking was not considered research (IRBNet #491165-4, 1/29/2019). Key strategies enlisted by the team to develop a retrospective list of trainees who participated in the program included the evaluation team’s participation records; payroll data (institutional records); identification of key records vital for tracking; and leveraging institutional and national records. Partnership trainee participation records were kept by the evaluation team starting in 2009; these data served as the foundation to retrospectively create a record of student participation. To backfill missing records from 2002 through 2008, the evaluation team worked with the Administrative Core’s Program Managers to obtain payroll records from 2002–2011 to identify students who were paid by the grant Cores and funded research projects at NAU and UACC. The Administrative Core also reviewed program archives, including past RPPR materials, to extract records of student participation. Next, the evaluation team worked with institutional officials at NAU and UACC to obtain student ID numbers and racial/ethnic data, which are critical for long-term outcome tracking. Finally, the team leveraged expertise at institutional data offices and utilized the National Student Clearinghouse (NSC) to obtain graduation and enrollment records for all participants (a task that is conducted annually to continuously update records offering validated data to support program reporting).

Annually, the NACP evaluation team works with the institutional research offices at NAU (Office of Strategic Planning, Institutional Research, and Analytics) and UA (University Analytics and Institutional Research) to request data through the National Student Clearinghouse (NSC). The NSC uses a service called “Student Tracker,” which uses a proprietary algorithm to match students to the NSC’s enrollment and degree information based on student name and date of birth [23]. The NSC provides abundant information about the student, including when and where students enroll, at what intensity (full or part-time), whether and when they earn a degree, and the major and type of degree earned [23].

**Figure 1:**
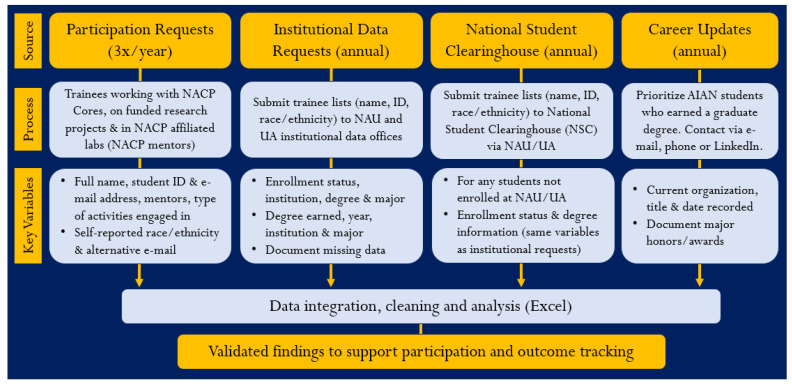
Tracking system inputs, data sources, and data collection periodicity.

*Implementation of a longitudinal trainee tracking and outcome system*: Once the team constructed a list of trainees engaged through partnership activities, the evaluation team developed a system to implement longitudinal participation and long-term tracking to inform program evaluation. The data management structure was driven by NACP’s output and reporting needs, which were also informed by CPACHE goals. Figure 1 outlines the data sources (participation requests, institutional data requests, National Student Clearinghouse (NSC), and career updates), describes the processes for obtaining records for each data source, and identifies key evaluation variables for each source. All data flows into an integrated Excel database for tracking validated findings to support participation and outcome tracking.

*Key variables in the NACP tracking database*: Due to our site’s reporting needs, we opted to create two separate tracking tabs, i.e., participation and outcomes (Table 3). Documenting records in two tabs results in a smaller number of duplications and makes reporting and analysis simpler. The participation tab aides the program in documenting the individual years (2002-present) that each person participated and includes trainee name (full, legal name); a simplified structure to indicate whether that trainee is Native American or Alaska Native (Y/N) to make filtering simpler; a detailed listing of each opportunity that a trainee participated in (by year); whether their combined participation included mentored research (including mentor names); and whether they specifically worked with an NACP-funded research project.

The outcome tab includes the following five key domains: (1) trainee details (name, student ID, alternative email, race/ethnicity); (2) key variables for optimizing reporting (mentored research, transition to a graduate program, and highest degree obtained); (3) degrees earned (allowing for multiple degrees); (4) enrollment status; and (5) current career status, career classification, and significant honors or awards. Career status is only documented when a student is not enrolled in an academic program. Career status records are updated annually through direct contact with the trainee, LinkedIn, or searches using known variables (such as last enrollment and degrees obtained to confirm trainees’ identities on professional organizations websites or institutions of higher education). We found that career information on social media sites (Facebook, etc.) tends to be outdated, thus those sites may only serve as a medium to contact the trainee (rather than serve as a data source). NIH parameters for trainee tracking were consistently vague (PARs simply required “tracking of participants”), until CareerTrac was adopted by the CPACHE and distinctions for which types of trainees should be included in CareerTrac. Thus, historically, NACP has not kept records to indicate trainees’ funding sources.

Key considerations for organizing tracking records: The NACP’s engagement opportunities for students (Table 2) are centered on the long-term goal of successfully transitioning Native American undergraduate students into advanced degree programs that will train them to address cancer health disparities in their communities. To that end, the partnership documents any degrees obtained after each trainee’s respective start date. Reporting prioritizes relevant degrees and career paths of trainees. To functionally track multiple degrees obtained in cancer-relevant careers (since an individual’s unique start date with NACP), the tracking sheet was developed to allow duplicates, meaning that for each degree obtained, a trainee has one record (row). Duplication of records makes it easier to count the total number of degrees obtained. The team is aware of the steps required for analysis, and we find that the system allows for streamlined analysis with basic technology needed and minimal financial resources. Because there are nearly 700 unique trainees (and growing), it is not reasonable to document enrollment status through time. Thus, enrollment records are overridden annually. Because of the volume of trainees and the focus of the partnership on Native Americans, the team chose to prioritize career outcomes for AIAN trainees who earned a master’s degree or higher. A variable for race/ethnicity reporting source was included to ensure compliance with Family Educational Rights and Privacy Act (FERPA) regulations. This allows a distinction between self-reported race/ethnicity and records obtained by institutional officials, as deidentified records can only be shared in aggregate if the race/ethnicity record came from institutional sources, whereas self-reported race/ethnicity can be used for program outreach to share professional development opportunities, for example.

## 3. Results

**Figure 2:**
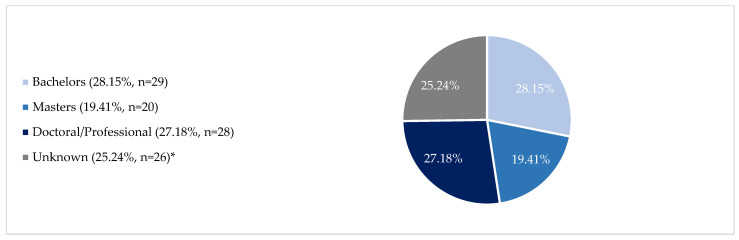
AIAN trainees in the pipeline: 2022 enrollment (*n* = 103). * Unknown: Record detail blocked: 6 at community/tribal colleges and 20 at another university (not NAU/UA).

*Collective participation and engagement*: Based on the successful development of this partnership, NACP is uniquely situated to advance cancer health equity for Native Americans and expand workforce development at all career stages. This paper focuses on the NACP’s engagement and subsequent outcomes for students (undergraduate through doctoral/professional programs). NACP created a strong pipeline for AIAN trainees who are engaged in an array of partnership activities (Table 2). In the partnership’s history (2022–2022), NACP engaged 686 students; over half (53%, 367) are AIAN. Given the focus of NACP on Native American and Alaska Native students, the remaining results center on AIAN trainees. During the current funding cycle, the partnership doubled AIAN student enrollment in doctoral or professional programs from 14 trainees (2019) enrolled in doctoral or professional programs to 28 trainees (2022). At the last evaluation checkpoint (2022), 103 AIAN trainees were enrolled, i.e., 29 in bachelor’s programs, 20 in master’s programs, 28 in doctoral/professional programs, and 26 were enrolled but had FERPA blocks on the enrollment detail, which means we could not obtain a specific degree program (Figure 2).

**Figure 3:**
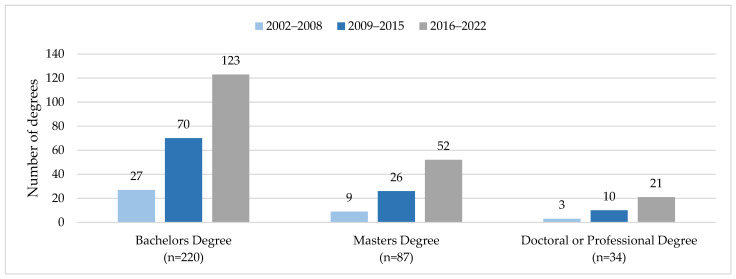
NACP AIAN trainees earned 341 degrees: 2002–2022 (*n* = 237 unique AIAN trainees) *. * Includes multiple degrees for individual trainees (for example, BS and MS).

*NACP impact on Native American and Alaska Native students*: Of the 367 AIAN trainees affiliated with the program, 237 students earned 341 degrees (some students earned more than one degree), 196 of which were earned in the past 7 years (Figure 3). The findings are organized over a 7-year period to evenly distribute units of measurement through time (Figure 3). Only degrees obtained after students became affiliated with NACP were included in tracking. Comparing two evaluation periods 2002–2008 and 2016–2022, the number of bachelor’s degrees attained by AIAN participants increased nearly 5-fold (27 to 123), master’s degrees nearly 6-fold (9 to 52), and doctoral degrees 7-fold (3 to 21). The most common bachelor’s degrees were in public health, biology, and biomedical science; the most common master’s degrees were in public health, biology, and chemistry. At a professional level, 10 AIAN trainees earned an MD, and 3 trainees earned a DPT, while the 21 individuals who earned a doctoral degree were from public health, American Indian studies, higher education, biochemistry, biology, biophysics, cancer biology, chemistry, clinical translational science, microbiology/immunology, and pharmacology/toxicology.

**Figure 4:**
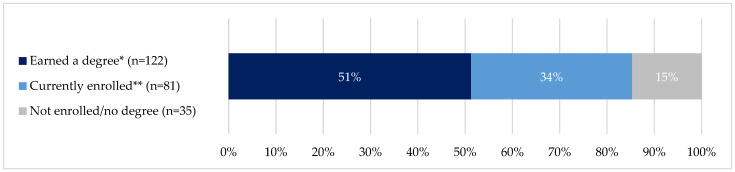
Outcomes among AIAN trainees who participated in mentored research: 2002–2022 (*n* = 238 trainees). * Earned any degree. Excludes students who were also enrolled in fall 2022. ** Includes 53 individuals who have already earned a degree.

*Outcomes for Native Americans and Alaska Natives who participated in mentored research:* Notably, 426 trainees were engaged in mentored research experiences 56% (n = 238) were AIAN. Moreover, 85% of AIAN trainees who participated in mentored research demonstrate strong outcomes with 51% having earned at least one degree and 34% were enrolled at the last checkpoint in 2022 (Figure 4).

**Figure 5:**
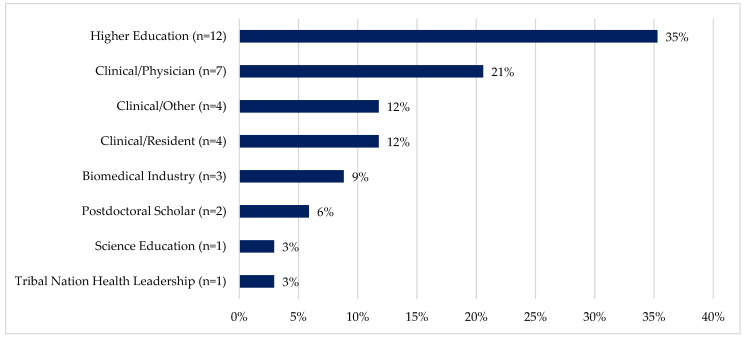
Career outcomes for AIAN trainees who earned a doctoral/professional degree: 2002–2022 (*n* = 34).

*Advancing cancer workforce capacity for Native Americans and Alaska Natives*: Career paths of AIAN trainees who obtained a doctoral or professional degree (n = 34) include postdoc or residency positions, working in higher education, clinical settings, biomedical industry, and science education, or notably, Tribal Health Department Leadership (Figure 5). After 22 years of CPACHE funding, the NACP Native trainees who started as undergraduates are finishing doctoral/professional programs and launching their cancer health equity careers. Notably, approximately 45% of AIAN doctoral/professional graduates were engaged in a professional career related to academic settings, including higher education (35%), science education (3%), or a postdoctoral fellowship (6%). Another 45% were in clinical care, with 21% in physician roles, 12% in resident roles, and 12% in other clinical roles.

## 4. Discussion

This manuscript provides insight into the development of a tracking system among a longstanding partnership (2002–2022) aimed at supporting the scientific workforce focused on cancer health equity among Native American populations. In the context of a CPACHE partnership, we document the growth of the program, the tracking approaches, and share AIAN trainee outcomes from the undergraduate to doctoral levels. As recommended by our Program Steering Committee early in the partnership, the NACP implemented a tracking system that leveraged team members from across the grant and engaged institutional leadership to develop a validated outcome tracking system. This underscores the value of the PSC to strengthen CPACHE programs when partnerships are responsive to recommendations. The growth in degree attainment suggests the value of long-term support for partnerships such as the NACP, further bolstered by the objectively validated outcome data utilized in tracking outcomes. Data from professional careers suggest that AIAN doctoral degree recipients are largely retained in academic and clinical settings, and the number of doctoral degree recipients accelerated over time.

This paper offers a resource for new partnerships or training programs looking to implement tracking systems or implement retrospective systems. A robust tracking system allows programs to evaluate key dimensions such as mentored vs. professional development, highest degree obtained, and transitions (undergraduate to graduate programs) with minimal missing data. This approach has direct implications for the program’s ability to demonstrate success to support RPPR, competitive renewals, and document program success. An approach only utilizing surveys or direct communication with program participants often results in poor response rates and missing data. Furthermore, self-reporting produces less reliable output than validated sources. The NACP tracking system consists of validated records from institutional data, NSC, and professional profile platforms such as LinkedIn. CareerTrac has potential to support CPACHE trainee tracking, which would be optimized by allowing trainees to self-report and update their own records in the system. This paper demonstrates the ability to develop a strong tracking system with low resources (funding and technology) that is sustainable without needing costly programming experts or other services.

We recommend that tracking data be coupled with student surveys, interviews, or other evaluation tools to contextualize students’ experiences of what influenced their ultimate cancer research career trajectories. NACP utilizes a number of evaluation instruments and processes to collect primary data, leveraging the CPACHE Research Education Evaluation Toolkit [24]. One such example is the NACP Annual Student Survey, adapted from the toolkit surveys to document experience and confidence with NACP-related research skills, science identity questions, NACP impact on career plans, challenges based on educational stage, feedback to improve programming, and mentor evaluation. These findings, paired with outcome data from the trainee tracking system, offer a robust understanding of program elements that support trainees and lead to their success in cancer-related careers. These results also inform program leaders of challenges and barriers faced by AIAN trainees, which may include academic departures for personal or familial needs, financial hardships (which were exacerbated during COVID-19), death, or academic setbacks. Many of these students do return to academia, and this situation further warrants long-term tracking because a straight trajectory of four years for undergraduates, two years for graduate school, and five years for a doctoral degree is often not followed.

Now that the training tracking system has been implemented, resulting in reproducible outcomes for degree, enrollment, and career outcomes, the partnership is starting to design a mixed-methods study to examine AIAN outcomes for students who participated for longer periods of time and had mentored research experiences. This study will leverage the tracking system capabilities presented in this paper with findings from semi-structured qualitative interviews to examine direct correlations between duration of training and insights about the most impactful opportunities and support for AIAN students engaged in the partnership. The proposed study offers a more robust analysis to determine whether certain factors (length of time engaged in mentored research) corelates with optimal trainee outcomes (degrees earned and careers in cancer health disparities).

## 5. Limitations

Several limitations should be noted. First, given the retrospective approach leveraging payroll records, data from early years (prior to 2009) exclude any trainees who may have directly been engaged by NACP but were not paid by the grant. NIH tracking criteria allows for inclusion of trainees engaged in cancer research through the partnership but may have been paid by another source (for example, working in the lab for course credit). Secondly, the documentation of honors and awards in the NACP tracking system is not exhaustive. While the partnership obtains updates about honors and awards in the annual student survey, the survey is only for active participants. Mentors and team members frequently share trainee’s successes (honors, etc.), but a more systematic way of documenting these achievements is needed.

The development of a custom database was also discussed but rejected due to costs for development and long-term maintenance. Ultimately, the team opted to use Excel due to staffing constraints and to ensure the sustainability of infrastructure. Although a relational database would allow robust reporting, records come from a multitude of sources (institutional records, NSC, participation tracking), requiring the team to manually enter all the data, making the idea of a custom relational database less appealing.

Limitations with CareerTrac were outlined for any programs that predated the NCI investment in CareerTrac for CPACHE sites, including additional work to track all trainees, including those who participated prior to CareerTrac utilization (in some cases, nearly 20 years of participants). We expect CareerTrac may be a more functional approach to tracking for newer partnership sites, although collecting validated data to report in CareerTrac remains a challenge. Finally, not all institutions of higher education may have access to validated data sources, such as the NSC, as it is a fee-based national dataset. This may limit some sites’ abilities to obtain validated degrees and enrollment records for trainees.

## 6. Conclusions

The ability to create reproducible findings for trainee outcome tracking using validated data is a requirement for workforce capacity-oriented programs. NCI leadership consistently cites the importance of tracking and evaluation to document the success of the CPACHE program and provide evidence to demonstrate the need for continued funding. NACP has demonstrated success in their ability to advance cancer workforce capacity for Native American and Alaska Native students through mentored research training opportunities, an array of professional development experiences, and a cancer health disparity curriculum. The process, key variables, and outcome measures serve as a model for programs to document their progress towards advancing workforce diversity for UR populations. NACP is proud of its ability to support Native students and to report an NACP graduation rate for AIAN students at 71% (almost twice as high as the national average of 38%), thereby increasing capacity for addressing Native cancer health disparities.

With continued CPACHE funding, the NACP team plans to innovate with approaches to training embedded in indigenous perspectives that we have honed with AIAN leadership and faculty, students, and the Community Advisory Board. Programming will expand across the Four Corners Region (AZ, CO, UT, and NM) to disseminate NACP’s training and mentoring approaches and increase the network for student placement. This approach will engage new partners, support trainees, and allow trainees to thrive in other academic environments to achieve the goal of expanding the AIAN cancer-focused research and health care workforce.

## Figures and Tables

**Table 1:** Shifting focus of CPACHE student engagement.

Funding Opportunity	Key Features Pertaining to Student Engagement (Language Adapted Directly from RFAs/PARs)
RFA-CA-02-005	Cancer Training and Career Development: Must focus on joint programs between MSI and Cancer Center(s) that place an emphasis on educating majority trainees to appreciate the issues associated with cancer disparities in minority populations (may lead to T32, K12, R25T). Cancer Education: Cancer education programs could focus on any effort to augment existing or create new curricula in the MSI and/or the Cancer Center (may result in an NCI R25E).
RFA-CA-09-501	Cancer Training (required): Create a cancer research training program (successful programs would result in T32, R25T, F31 awards, K awards, and research supplements for trainees). Cancer Education (optional): Augment existing curricula or create new curricula (may result in an R25 submission).
RFA-CA-11-001	Cancer Training (required): Emphasize the training of underrepresented (UR) investigators and students and understanding of the issues. associated with cancer disparities in underserved populations. Cancer Education (required if curriculum development is an inherent part of the training program) May result in an R25 submission.
PAR-18-361	Research Education Core (required): Supports joint research education programs between the partner institutions that offer research experiences, curriculum development, or both (linking faculty and students).

**Table 2:** Overview and classification of NACP student engagement.

Type of Engagement	NACP Research, Training, and Education Opportunities	Examples (Not Exhaustive)
Mentored Research	Conducting cancer-related research with NACP affiliated investigators/faculty	NACP research projects and summer research exchange (NAU/UACC)
	Transitions to college or university	Summer Transitional Enrichment Program (STEP) (funded as a Bridges T34)
	Summer internships at other institutions	National internships (NIH)
	Individualized career development plan	UACC 1 credit course (Individual Development Planning for a Biomedical Career (MCB 396K))
Professional Development	Participation at national conferences	AISES, SACNAS, ABRCMS, and AACR *
	AIAN role model speaker series	My journey/role model programs and career path exploration
	Graduate programs primer	Graduate college programs, lab tours, and AIAN student panels
	Science writing and communication	Science communication sessions at the summer research conference, resume/CV development, press release, and abstract writing
	AIAN cancer health disparity (CHD) education modules	CHD curriculum on D2L (UACC) and Canvas (NAU) platforms

* American Indian Science and Engineering Society (AISES), Society for Advancement of Chicanos and Native Americans in Science (SACNAS), Annual Biomedical Research Conference for Minoritized Scientists (ABRCMS), and American Association for Cancer Research (AACR).

**Table 3:** Participation and annual outcome-tracking database variables.

Participation Variables	Outcome Variables	
Student name AIAN (Y/N) Ethnicity and race Specific program Mentored research (Y/N) Mentor name(s) Full/Pilot project related	Trainee name (first last) Year started with NACP Student ID number Race/Ethnicity Reporting Source (race/ethnicity) Mentored research (Y/N) Transition to graduate program Highest degree obtained Highest degree obtained (detail) Degree earned (level) Degree earned (detail) Degree earned (discipline) Year degree obtained Institution where degree earned	Currently enrolled? (Y/N) Enrollment Institution—category Enrollment Institution—other Enrollment degree category Enrollment degree type Enrollment major Enrollment updated Career type (NIH categories) Current position and employer Date career record last updated Source for career position Email (alternative) Phone number Special awards/honors

## Data Availability

Restrictions apply to the datasets. The datasets presented in this article are not readily available due to FERPA protections and technical limitations to de-identify the records.
